# Free Energy and Flexibility Analysis of Autoinhibited Human BRAF

**DOI:** 10.1021/acs.jcim.5c03126

**Published:** 2026-04-21

**Authors:** Jeremy O. B. Tempkin, Fikret Aydin, Sebnem Essiz, Yue Yang, Timothy S. Carpenter, David E. Durrant, Deborah K. Morrison, Helgi I. Ingólfsson, Frederick H. Streitz, Dwight V. Nissely, Felice C. Lightstone, Xiaohua Zhang

**Affiliations:** † Physical and Life Sciences Directorate, 4578 Lawrence Livermore National Laboratory, Livermore, California 94550, United States; ‡ Faculty of Natural Sciences and Engineering, Department of Molecular Biology and Genetics, 52974 Kadir Has University, Fatih 34083 Istanbul, Turkey; § Laboratory of Cell and Developmental Signaling, Center for Cancer Research (CCR), National Cancer Institute (NCI), Frederick, Maryland 21702, United States; ∥ Computing Directorate, Lawrence Livermore National Laboratory, Livermore, California 94550, United States; ⊥ NCI RAS Initiative, Cancer Research Technology Program, Frederick National Laboratory for Cancer Research, Frederick, Maryland 21702, United States

## Abstract

The RAF serine/threonine protein kinases function as direct effectors of RAS in the intracellular transmission of extracellular growth signals, and they are key targets for drug discovery, given the high incidence of oncogenic mutations in RAF and other components of this signaling pathway. In its inactive state, RAF is held in an autoinhibited conformation in the cytosol through a combination of intramolecular interactions and binding to a regulatory 14–3–3 protein dimer. Activation of RAF is initiated by its interaction with membrane-localized GTP-bound RAS, which induces conformational changes that release RAF from its autoinhibited state. However, the molecular mechanisms governing RAF activation remain incomplete, largely due to the challenges in experimentally capturing the intermediate conformational states in this process. To address this gap, we developed a comprehensive all-atom model of BRAF based on existing cryo-EM structures. Using this model, we performed extensive molecular dynamics simulations to evaluate the stability and free energy landscape of autoinhibited BRAF in solution. Our analysis reveals conformational flexibility within the autoinhibited complex, suggesting that this dynamic behavior may play a role in facilitating BRAF activation upon engagement with the membrane-bound RAS.

## Graphical abstract

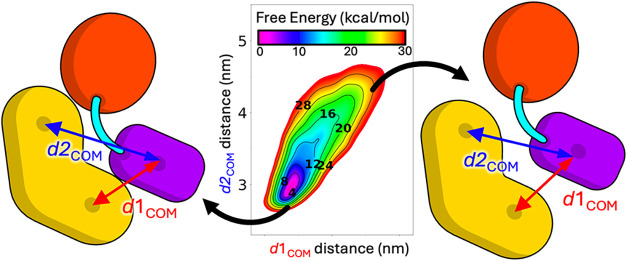

## Introduction

The RAF family of kinases, comprising ARAF, BRAF, and CRAF, occupies a central position in the MAPK signaling cascade, transmitting extracellular signals from receptor tyrosine kinases to downstream effectors such as ERK.^1^ Among these isoforms, BRAF has gathered considerable attention due to its frequent mutation and dysregulation in various cancers, including melanoma, colorectal cancer, and thyroid cancer.^2^

Structurally, BRAF contains several distinct domains, including an N-terminal RAS-binding domain (RBD), a cysteine-rich domain (CRD), and a C-terminal kinase domain (KD). A 14–3–3 dimer (14–3–3_2_) binds to phosphorylated serines 365 and 729 (referred to here as 14–3–3 (S365) and 14–3–3 (S729), respectively), stabilizing the autoinhibited conformation and occluding both the dimer interface region of the KD and the membrane-binding region of the CRD. Upon growth factor engagement of receptor tyrosine kinases (RTKs), RAS becomes activated and recruits BRAF to the plasma membrane via high-affinity interactions with the RBD. Once recruited, BRAF undergoes structural modifications. Release of 14–3–3 from the serine 365 site and the subsequent dephosphorylation of this site by the SHOC2-MRAS-PP1 complex enable CRD to interact with membrane lipids and RAS to facilitate RAF dimerization for full KD activation.^1,2^

Additionally, in the cryo-EM structure of the autoinhibited BRAF complex (PDB 7MFD),^3–5^ key ionic bond-forming residues on the RBD that mediate high-affinity RAS binding are largely exposed. However, structural overlay of RAS onto the complex reveals steric clashes between RAS and 14–3–3, indicating that conformational rearrangements of the RBD, 14–3–3, or both are likely required to permit full RAS engagement. Despite these insights, the precise mechanistic details governing the transition from the autoinhibited BRAF-14–3–3_2_ complex to signaling-competent conformations remain poorly understood. Furthermore, the exact sequence of structural events leading to RAF activation is still unclear. A deeper mechanistic understanding of these transitions and structural states could reveal novel targets for therapeutic intervention.

To investigate the intrinsic flexibility of RAF and the initial conformational changes required to relieve autoinhibition upon RAS binding, we constructed a comprehensive all-atom (AA) model of the autoinhibited BRAF:14–3–3_2_ complex in solution, based on a previously reported cryo-EM structure.^3^ Our model encompasses BRAF residues 156–738, including two large intrinsically disordered regions that connect the cysteine-rich domain (CRD) to the kinase domain (KD) and surround the pS365 binding site for the 14–3–3 dimer (14–3–3_2_). Using this model, we performed extensive AA molecular dynamics (MD) simulations to characterize the conformational landscape around the fully autoinhibited state in solution. We applied essential dynamics analysis and state-of-the-art free energy sampling methods to quantify both the lowest vibrational models near the autoinhibited state and the relative free energy barriers associated with specific interdomain interactions known to be implicated in BRAF activation. Our results reveal that the lowest-frequency motions correspond to a hinge-like movement that displaces the KD away from the CRD and the 14–3–3_2_ dimer interface. Furthermore, the KD interface with the CRD is considerably more flexible than the interface between the 14–3–3_2_ dimer and the CRD, having a free energy barrier to disruption comparable to that of the RBD:14–3–3 interface. These findings suggest that the motions of the KD domain may constitute a critical step in the BRAF activation process by facilitating the disruption of autoinhibitory interactions to promote RAF dimerization and activation.

## Results and Discussion

### Structural Heterogeneity of the Disordered Region Loops I and II

Loops I and II, totaling approximately 150 residues in length (Figure 1), are disordered in solution and are likely unresolved in the cryo-EM structure,^3^ with the exception of the ∼10 residue section around the serine 365 site. To assess the impact of loop placement on the conformational landscape of autoinhibited BRAF, we generated a large ensemble of initial configurations through randomized positions of loops I and II, followed by relaxation and sampling of the conformational landscape via unbiased MD simulations (Figures 1 and S3). Although the initial positions of the loops are qualitatively similar, the relaxed ensemble sampled from the unbiased MD simulation shows that the loops are flexible, sampling the full space around the BRAF complex, with no apparent high affinity for other domains within the autoinhibited complex. Plotting the average density of loop occupations at low, medium, and high threshold values shows that the loops sample a broad area around the complex and only have high occupancy at the positions where the loops are stably bound to the complex (Figure S3). Furthermore, the relaxation of loops I and II shows that the ensemble is less extended than in the initial placement (Figure S3).

**Figure 1:**
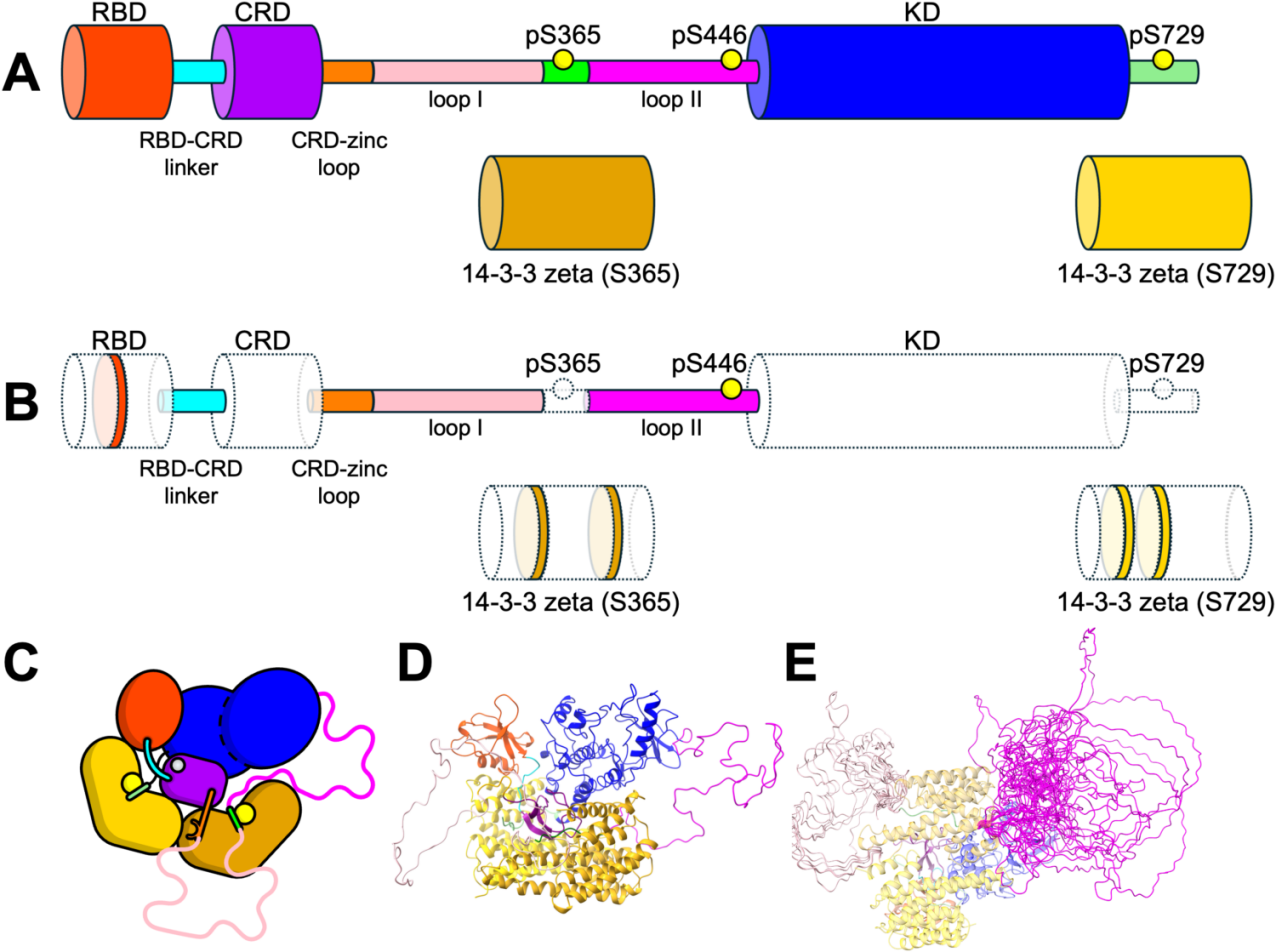
Atomistic model of the complete BRAF-14–3–3_2_ complex in solution. (A) Cartoon illustration of the BRAF-14–3–3_2_ complex. The BRAF:14–3–3_2_ complex consists of 11 distinct domains: the RBD (residues 156 to 227), RBD-CRD linker (residues 228 to 234), CRD (residues 235 to 273), CRD-zinc loop (residues 274 to 281), loop I (residues 282 to 359), pS362 region (residues 360 to 370), loop II (residues 371 to 456), KD (residues 457 to 723), pS729 region (residues 724 to 738), 14–3–3 zeta (S365), and 14–3–3 zeta (S729). Thicker tubes indicate globular domains (RBD, CRD, KD, and both the 14–3–3 protomers in red, purple, blue, gold, and yellow, respectively), and thinner tubes indicate linker regions or disordered sections (in cyan, orange, pink, green, and magenta). Phosphorylation sites at residues S365, S446, and S729 included in our model are indicated by the yellow circles. (B) Cartoon of the BRAF-14–3–3_2_ complex illustrating which sections of the structure were modeled into the original cryo-EM structure (PDB 7MFD). Domains and residues that are resolved in the cryo-EM structure are shaded (white fill with dashed lines), and sections that were modeled in are colored. (C) Cartoon of the autoinhibited BRAF-14–3–3_2_ complex showing what domains are in contact using the canonical coloring for each domain. (D) Example of the initial structure of the BRAF-14–3–3_2_ complex after the initial simulation relaxation using unbiased MD. (E) Illustration of the library of initial disordered loops I and II built with the MOE loop modeling procedure. Loops I (pink) and II (magenta) are shown superimposed on a single BRAF-14–3–3_2_ complex structure aligned to the 14–3–3_2_. View in panel (E) is from 90° rotation from view in panel (D).

To confirm the proper equilibration of the loop configurations, we plotted the radius of gyration (RoG) and RMSD of the disordered loops (Figure S4) starting from their initial positions (Figures 1 and S3). All trajectories appear stable at an RMSD of about 25–40 Å for loop I and 10–25 Å for loop II after ∼200 ns of simulation. Outside of the relaxation of loops I and II, no other significant conformational changes in the folded domains were observed in the unbiased simulations, indicating that the overall model is energetically stable around the autoinhibited state.

### Principal Components in the Essential Dynamics Analysis Reveal the Inherent Flexibility of the RBD and KD in the Autoinhibited Solution Structure

To investigate the conformational flexibility of the BRAF-14–3–3_2_ complex in solution, we computed the essential dynamics around the autoinhibited state over a large ensemble of structures derived from our library of unbiased MD simulations (Figure 2). We focused on the first two principal component analysis (PCA) modes, which together represent ∼36% of the observed fluctuations around the autoinhibited state. Extending our analysis to the first five modes accounts for ∼52% of the observed fluctuations (Figure S5A). In the first mode (Figure 2A,C), the RBD and KD open in a hinge-like motion around the point where the CRD interfaces with the 14–3–3 dimer. Simultaneously, the CRD moves outward from this hinge region. In the second mode (Figure 2B,D), the RBD and KD exhibit a twisting motion, again centered at the CRD:14–3–3 interface, resulting in the RBD and KD rotating away from each other. In both modes, the RBD and KD are the most mobile regions of the complex, while the 14–3–3_2_ dimer remains comparatively rigid. In the third mode, each domain exhibits more locally correlated motions (Figure S5E); however, the RBD and KD are still the most mobile with the 14–3–3_2_ exhibiting a twisting motion with respect to the rest of the complex (Figure S5B). The fourth mode is dominated by the KD moving away from the RBD and 14–3–3 dimer (Figure S5C), whereas the fifth mode is dominated by a twisting motion of the RBD (Figure S5D).

**Figure 2:**
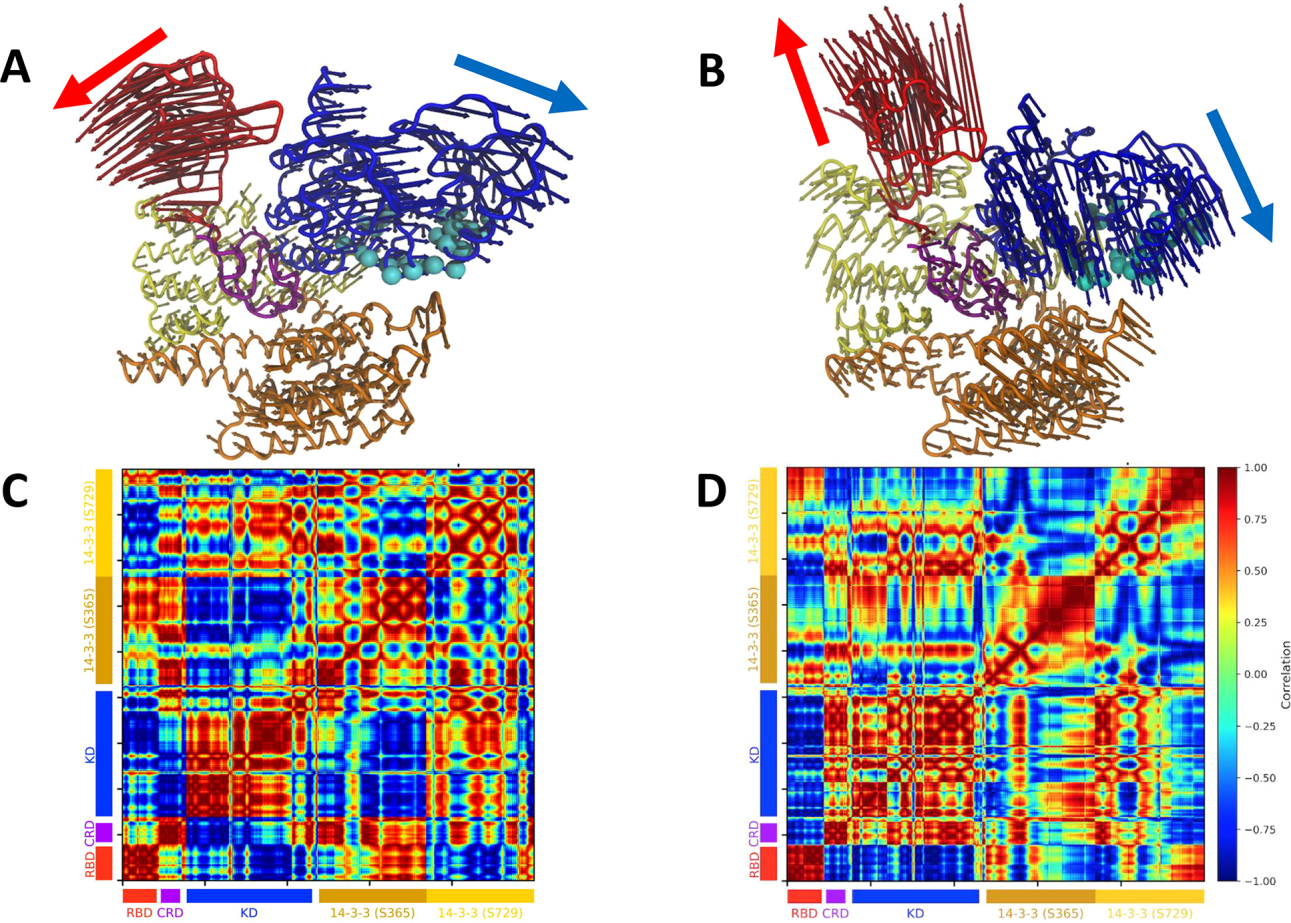
PCA analysis of the autoinhibited BRAF:14–3–3_2_ complex. The proteins/protein domains are shown as follows: RBD: red, CRD: purple, KD: blue, 14–3–3 (S365): gold, and 14–3–3 (S729): yellow. Residues that form the KD-to-KD interface in the dimeric BRAF structure (PDB: 7MFF) are displayed as cyan spheres. (A) First PCA mode visualization and (C) cross-correlation map of the mode. (B, D) Same for the second PCA mode. The colored arrows indicate the primary motions of each mode.

To further characterize these motions, we computed the normalized cross-correlation between domain movements in the first two modes (Figure 2C,D). Interestingly, the CRD displays opposing behavior between the first and second modes: in the first mode, the CRD motion is anticorrelated with that of the KD, whereas in the second mode, it moves in concert with KD. These results indicate that equilibrium fluctuations around the autoinhibited state are primarily driven by motions involving the RBD and KD.

Finally, to assess whether the conformational flexibility around the autoinhibited state is related to the conformational changes required for the KD to transition between the monomeric and dimeric states, we compared these motions to the structural differences observed between the monomeric (PDB: 7MFD) and dimeric (PDB: 7MFF) cryo-EM structures of BRAF.^3^ We observed a qualitative similarity between the structural transformation required to convert the dimeric state to the monomeric state and the motion described by the second PCA mode (Figure S6). Additionally, in the first four modes, the 14–3–3 (S365) domain moves away from the residues that form the KD-to-KD interface in the dimeric BRAF structure (PDB: 7MFF), suggesting that these motions may facilitate the KD dimer-to-monomer transition (Figure 2A, B).

Overlaying RAS onto the autoinhibited BRAF structure reveals a steric clash between RAS and the 14–3–3 dimer when high-affinity ionic bonds are formed between the RBD and RAS.^3^ This observation suggests that conformational flexibility within the complex is likely required to accommodate full RAS engagement. Consistent with this expectation, the RBD exhibited a relatively high mobility in our unbiased MD simulations. However, a more unexpected finding was the substantial motion observed in the KD, despite the complex being in an autoinhibited state. The relative flexibility of the KD observed here is consistent with recent studies showing that MEK binding suppresses BRAF activation by stabilizing the autoinhibited conformation.^37^ PCA analysis of the simulation trajectories revealed that the dominant motions around the autoinhibited conformation involve both the RBD and KD, whereas CRD and the 14–3–3 dimer remain comparatively stable. These results raise the intriguing possibility that KD mobility may play a functional role early in the RAF activation process, potentially priming the complex for conformational rearrangement upon RAS engagement.

### Free Energy Landscape of BRAF in Solution Shows that the Relative Flexibility of the KD Is Similar to the RBD

As the BRAF:14–3–3_2_ complex reorients and begins to disassemble, the conformational changes leading to BRAF activation are most likely to proceed through the lowest free energy path connecting the fully autoinhibited complex state to the monomeric predimer state. In the earliest stages of this process, the interdomain interactions observed in the autoinhibited state are likely to remain intact. Thus, these transitions are expected to be dominated by the intrinsic interdomain free energies surrounding the autoinhibited state. Therefore, mapping this landscape, particularly the free energy associated with interdomain motion in solution, may provide valuable insights into the likely sequence of conformational changes that occur following RAS engagement, shedding light on the earliest events in the BRAF activation process.

To further characterize the stability of the BRAF autoinhibited state in solution, we mapped the free energy landscape of the complete autoinhibited BRAF:14–3–3_2_ complex in solution using extensive one-dimensional umbrella sampling simulations.^24^ We selected six interdomain coordinates that span critical interactions between the RBD, CRD, KD, and both 14–3–3 protomers (Figure 3): (1) RBD to KD, (2) RBD to 14–3–3 (S729), (3) CRD to 14–3–3 (S729), (4) 14–3–3 (S729) to KD, (5) 14–3–3 (S365) to KD, and (6) CRD to KD. Each coordinate was defined as the distance between the centers of mass (COM) of all Cα atoms within the respective domains. These coordinates were chosen to capture the key interdomain motions hypothesized to initiate the activation process (Figure 3) based on the analysis of the essential dynamics of the complex around the autoinhibited state (Figure 2). The first two coordinates describe the positioning of the RBD with respect to the 14–3–3 (S729) protomer and KD. Coordinates 3–6 describe the positioning of the KD and the CRD with respect to the 14–3–3 dimer. Each of these coordinates was selected manually to cover the possible directions of interdomain flexibility in the complex. To account for the conformational heterogeneity of the disordered loops, we repeated the umbrella sampling procedure for each of the 27 loop conformers, resulting in a combined simulation time of over half a millisecond. While loops I and II are dynamic (Figure S3), metastable interactions between the loops and structured domains can bias the free energy estimates if simulations are too short or limited in number. Our ensemble-based approach mitigates this risk and better captures the broader thermodynamic behavior of the autoinhibited complex.

**Figure 3:**
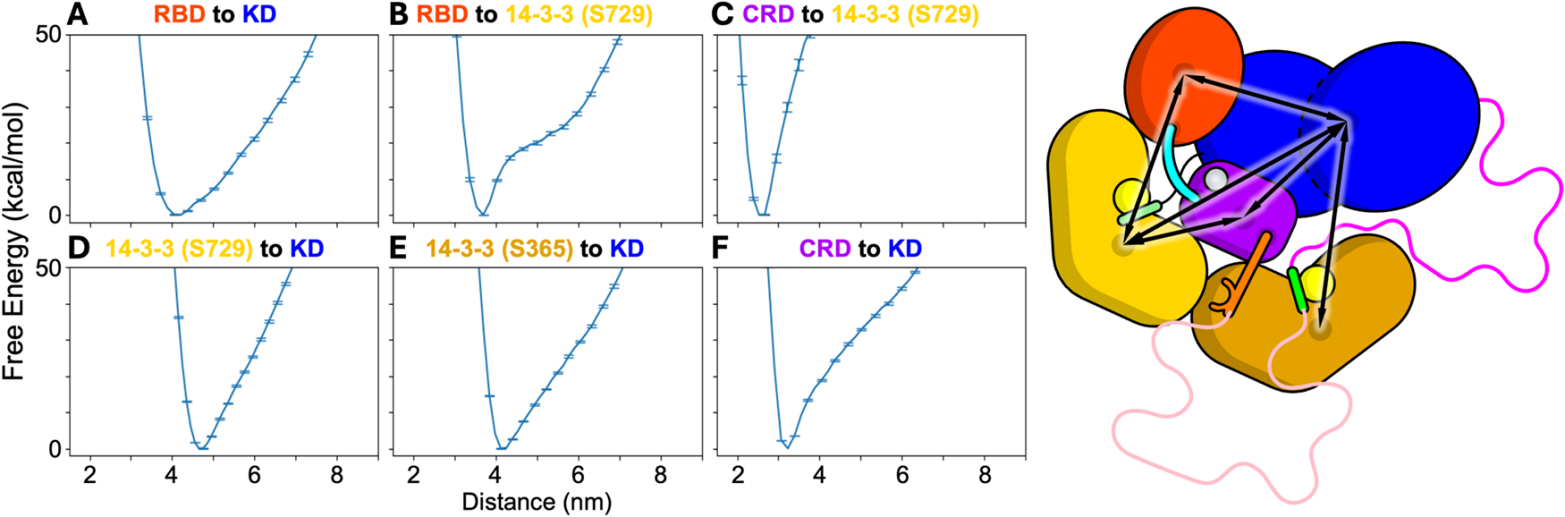
Free energy surfaces of interdomain distances in the autoinhibited BRAF:14–3–3_2_ complex computed by 1D umbrella sampling. Interdomain distances are computed as the distance between the COM of all Ca atoms in each domain for the following pairs of domains: (A) RBD to KD, (B) RBD to 14–3–3 (S729), (C) CRD to 14–3–3 (S729), (D) KD to 14–3–3 (S729), (E) KD to 14–3–3 (S365), and (F) KD to CRD. The free energies are estimated by grouping samples from similar windows across each of the 27 independent 1D umbrella sampling simulations, which were started from each loop structure. The estimate of the free energies at each window are computed using the WHAM. Error bars are estimated from the MC bootstrapping procedure provided in WHAM software using 100 iterations, indicating the 95% confidence interval around the mean. A cartoon of the autoinhibited BRAF:14–3–3_2_ complex is shown indicating all the interdomain distances (right).

The 1D free energy profiles indicate that the KD and RBD exhibit the greatest conformational flexibility around the autoinhibited state, consistent with the normal-mode analysis of the complex (Figures 2 and 3). The free energy profiles along the RBD to the 14–3–3 (S729) coordinate show that the RBD is bound to the 14–3–3 (S729) protomer with an ∼20 kcal/mol energy barrier (Figure 3B). Between ∼4.3 and ∼5.8 nm, the RBD dissociates from its native interface with 14–3–3 (S729) but remains tethered to the CRD via a short 6-residue disordered linker. The rise in free energy over this distance range corresponds to the progression extension of this linker. Beyond ∼5.8 nm, the linker between the RBD and CRD is fully extended, and further separation of the domains requires disruption of interactions between the CRD and the 14–3–3 dimer. In the autoinhibited state, the RAS binding interface of the RBD is occluded due to interactions with 14–3–3 (S729), which involve key basic residues known to be critical for RAS recognition (R158, R166, K183, and R188).^3^ The total number of interdomain contacts formed between these four critical residues in the RBD and the 14–3–3 (S729) domain decreases steadily as the domains are pulled apart, indicating a progressive release of the RAS binding surface and suggesting that this coordinate could be critical for exposing the RAS binding site on the RBD. Furthermore, the free energy profile for the RBD to the KD coordinate shows that the RBD position with respect to the KD is relatively stable, likely due to the persistent binding of the RBD to 14–3–3 (S729) in these simulations. Snapshots of the BRAF complex in its extended form are provided in Supporting Figure S6 and full structures are available to download online.

The free energy profiles for the pulling directions between the CRD and both 14–3–3 protomers show that these interactions are stably maintained in the autoinhibited state, with a high energetic cost to disrupt them. Furthermore, the coordinates between the KD and both 14–3–3 protomers are similarly stable. However, the energy gradient for separating the KD from the 14–3–3_2_:CRD complex is less steep than that observed for the CRD:14–3–3_2_ interactions, suggesting a somewhat more flexible interface. In contrast, the KD-to-CRD direction shows that the KD is bound to CRD with an energy barrier of ∼20 kcal/mol (Figure 3F), which is comparable to the free energy required to dissociate the RBD from the 14–3–3 (S729) protomer. Beyond a separation of ∼4 nm, further displacement of the KD requires the extension of intrinsically disordered loops I and II.

### Disengaging the RBD from 14–3–3 (S729) Increases the Flexibility of the CRD-to-14–3–3_2_ Interface

For membrane-bound RAS to fully engage the RBD of BRAF, the native interface between the RBD and the 14–3–3 (S729) protomer must be at least partially disrupted.^3^ To investigate how disruption of the RBD–14–3–3 (S729) interface affects the conformational free energy landscape of BRAF, we performed a series of two-dimensional (2D) metadynamics simulations starting from two distinct conformational states: one in which the RBD forms a native interface with the 14–3–3 (S729) protomer (“RBD-engaged” state) and one in which this interface is broken (“RBD-disengaged” state) (Figure 4).

**Figure 4:**
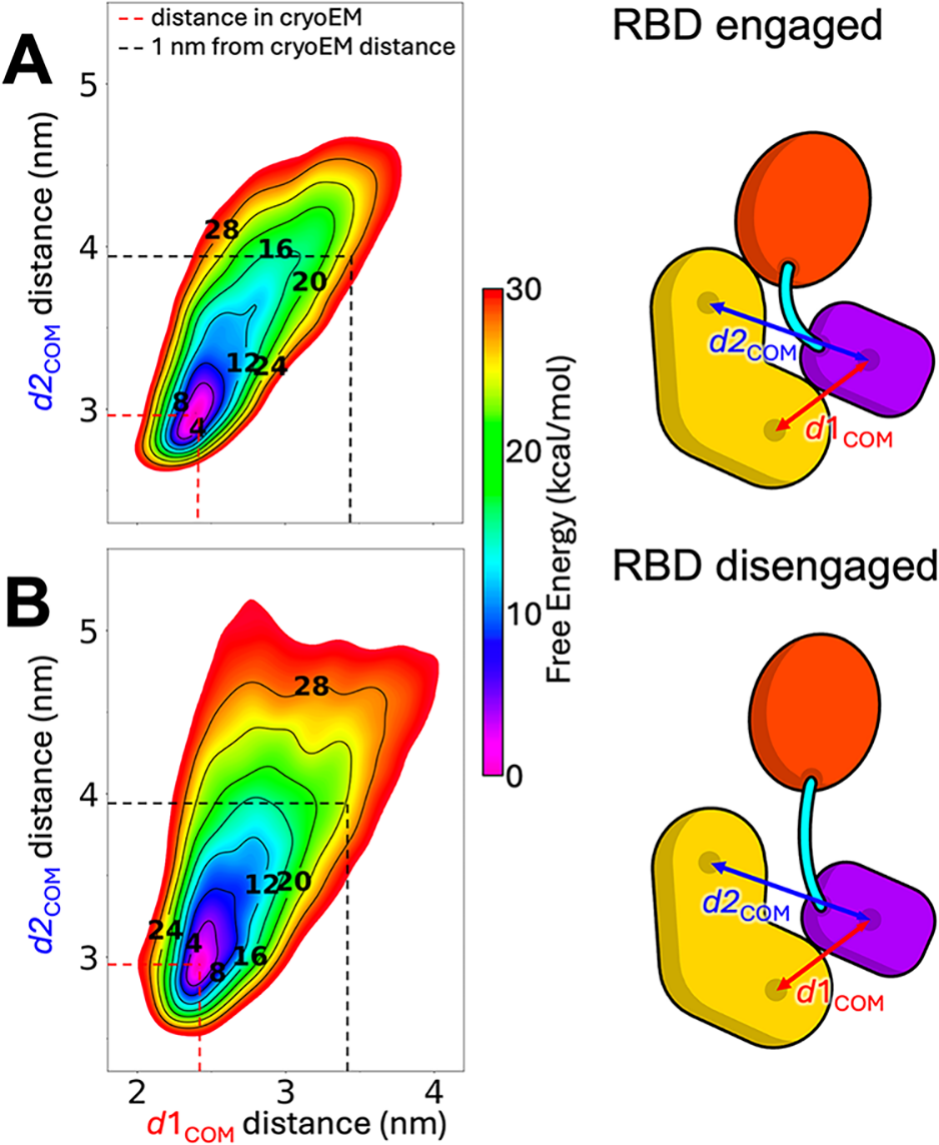
Binding of RAS influences the stability of the 14–3–3 (S729)-CRD interface. 2D potential of mean force (in kcal/mol) obtained by averaging multiple independent metadynamics simulations for understanding the conformational mobility of the 14–3–3 (S729)-CRD interface when RBD is bound (A) or unbound (B) to 14–3–3 (S729). The collective variables (CVs) d1COM and d2COM indicate the COM distances between the first half of 14–3–3 (S729) (the region closer to CRD) and CRD and between the COM of the second half of 14–3–3 (S729) (the region closer to RBD) and CRD, respectively.

The resulting 2D potential of mean force (PMF) plots (Figure 4) capture the motion of the 14–3–3 (S729) protomer relative to the CRD along two collective variables (CVs). As described in the Methods Section, CV1 represents the center-of-mass (COM) distance between the CRD and the N-terminal half of the 14–3–3 (S729) protomer (proximal to the CRD), while CV2 tracks the COM distance between the CRD and the C-terminal half of the same protomer (closer to the RBD). These CVs were chosen to bias the system toward conformations in which the 14–3–3 protomer moves away from the CRD, allowing the exploration of higher energy, partially dissociated states. Although the interactions at the CRD–14–3–3 interface were disrupted during sampling, the 14–3–3 protomer remained tethered to the phosphorylated S729 residue.

The free energy landscapes reveal that the motion of the 14–3–3 (S729) protomer is more restricted in the RBD-engaged state than in the RBD-disengaged state (Figure 4A,B). In the engaged configuration, large conformational rearrangements are energetically disfavored, confining the 14–3–3 protomer to a narrower region of the conformational space. This rigidity is likely stabilized by a network of hydrogen bonds formed at the RBD–14–3–3 interface, as described in previous structural studies.^3^

In contrast, the RBD-disengaged state permits greater conformational freedom of the 14–3–3 protomer, highlighting how the RBD displacement may be a critical step in destabilizing the autoinhibited conformation. These findings suggest that RAS binding, by trapping the RBD in a disengaged state, could facilitate the conformational transitions necessary for BRAF activation.

## Conclusions

To characterize the conformational flexibility of autoinhibited BRAF, we constructed a comprehensive all-atom model of the BRAF:14–3–3_2_ complex and investigated its dynamics through extensive molecular dynamics simulations and free energy calculations. Our analysis of the essential dynamics and interdomain free energy landscapes confirms that the BRAF:14–3–3_2_ complex is stable in its autoinhibited state.

Among the components of the complex, only the disordered loops I and II exhibit significantly dynamic behavior (Figures 1 and S3). Quantification of interdomain motions revealed a hinge-like, low-frequency movement of both the RBD and KD away from the CRD and the remainder of the complex (Figure 2). These findings are consistent with the lower free energy barriers associated with the displacement of the RBD and KD (Figure 3). The relatively high mobility of the RBD is expected, as the RAS-binding interface on the RBD is partially occluded in the autoinhibited structure,^3^ necessitating RBD displacement for full RAS engagement. Moreover, RAS binding has been experimentally shown to enhance the BRAF activation. Notably, disengagement of the RBD from the 14–3–3 (S729) protomer also increases the conformational mobility of the 14–3–3 (S729) protomer relative to the CRD (Figure 4), thereby reducing energetic barriers for subsequent steps in the release of autoinhibition.

Surprisingly, the KD also demonstrates a relatively low barrier for displacement and a similar low-frequency, hinge-like motion away from the CRD and 14–3–3_2_. This finding suggests that the KD movement may play an active role in the early stages of BRAF activation. This mechanism is supported by recent experimental studies showing that stabilizing the KD—through either MEK binding^37^ or BRAF inhibitors^38^—can suppress BRAF activation by reinforcing the autoinhibited conformation.

Taken together, our findings support a model in which BRAF autoinhibition is maintained by a network of stabilizing interdomain interactions. While stable on short time scales, these interactions may fluctuate or weaken over longer periods, particularly in the presence of RAS or other modulatory factors. Such external influences could either promote reengagement of the autoinhibited state or facilitate transitions toward activation, providing mechanistic insights into how RAF regulation is dynamically controlled at the molecular level.

## Methods

### Building the Complete Autoinhibited BRAF Model

We constructed our model of the autoinhibited BRAF:14–3–3_2_ complex based on the recent cryo-EM structure of the BRAF:14–3–3_2_:MEK complex (PDB: 7MFD).^3^ To build the model for MD simulations, several additions and modifications were made, as described below. First, MEK was removed from the complex to reduce the system complexity and computational cost (further discussed in the Results and Discussion Section). ATP and its coordinating Mg^2+^ ion were then placed using Molecular Operating Environment (MOE)^6^ (v2022.02) with PDB 6U2G (chain B) serving as the template (Supporting Information Figure S1). Adjustments to the contacting amino acids were made to reflect the ATP and Mg^2+^ binding in PDB entry 6U2G, followed by a short energy minimization to relax the ATP-Mg complex. We also restored a missing residue at the N-terminus of the 14–3–3 proteins, near the interface of the 14–3–3 dimer, as well as two residues in the loop region (E70, G71). The C-terminus was capped without reconstructing the 15 missing residues (D231 to N245) as this region is distant from the interface. To complete the coordination of one of the zinc-binding sites in the CRD, we added a water molecule to fulfill the zinc coordination requirements and adjusted its placement to ensure the proper coordination of the H235 residue (Figure S2). The remaining missing residues (listed in Table S1) were added using the MOE protein structure preparation model using the default parameters. We added phosphorylation sites to the model at S365, S446, and S729, as these sites are phosphorylated in the quiescent state of BRAF.^4,7^ We elected to use a charge of −1 for each phosphorylation site.

Finally, we added the missing intrinsically disordered regions that connect the C-terminus of the CRD to the N-terminus of the KD (Figures 1 and S3), which were unresolved in cryo-EM structure 7MFD. This linker is divided into two distinct segments, named here loop I (residues 282 to 359) and loop II (residues 371 to 456). Loop I connects the C-terminus of the CRD to the S365 regulatory site, while loop II connects the C-terminal side of the S365 site to the N-terminal side of the KD (Figure 1). To incorporate these disordered regions into our model for MD simulations, we generated a randomized ensemble of initial loop placements using the following procedure. First, we modeled an ensemble of putative loop conformers into the cryo-EM structure using MOE Loop Modeler with default de novo loop building parameters. For each loop region, we generated 100 independent loop conformers. From these, we manually selected a representative subset based on conformational diversity and energy score, yielding nine conformers for loop I and three conformers for loop II. We then combined all possible loop I and II pairs, resulting in an initial library of 27 distinct structural models (Figures 1E and S3).

For each initial conformer, we initialized and simulated five independent replicas starting from the same initial configuration of unbiased MD simulations using the AMBER18 package.^8–10^ All input files were generated using CHARMM-GUI^11,12^ with standard settings. All MD simulations were then performed using the CHARMM36m force field^13^ with a 4 fs time step (enabled by hydrogen mass repartitioning^14^) unless otherwise noted. The TIP3P^15^ water model was used, with water constraints applied via SETTLE^16^ and all other hydrogens constrained using SHAKE.^17^ These unbiased AA simulations were run with mixed-precision (SPFP^18^) AMBER18, following initial topology construction and minimization with GROMACS (v2019.6)^19^ and format conversion with the Gromber tool of ParmEd from AmberTools 19.^20^ Each individual MD simulation within this set was simulated for a minimum of 500 ns each. This procedure generated a library of 135 independent simulations containing a total of 73.2 μs of simulation data.

### Essential Dynamics Analysis of Unbiased Simulations

As described in the model-building section, we generated 27 distinct structural models of the autoinhibited BRAF complex, representing all possible combinations of our randomized initial ensemble of loop I and loop II conformations. We used a library of 135000 frames from the unbiased MD simulations by selecting the final 1000 frames (last 100 ns of 500 ns) from each of the 135 unbiased MD simulations described above. For the five replicas of each different loop combination, we checked the radius of gyration (RoG) and in the last 100 ns, a compact structure had been reached for all simulations. The radius of gyration (Rg) and root-mean-square deviation (RMSD) were calculated using the analysis routines implemented in the MDAnalysis package.^21^ The Rg quantifies the spatial distribution of atoms around the center of mass of a protein and measures how compact the structure is. In MDAnalysis, it is computed as the mass-weighted root-mean-square distance of atoms from the center of mass. The RMSD measures the structural deviation of a configuration relative to a reference structure. In MDAnalysis, RMSD is computed as the mass-weighted root-mean-square distances between the corresponding atoms in the superimposed structures after optimal superposition to remove rotational and translational degrees of freedom. In both analyses, only Cα atoms of the protein domains were used, as the side-chain mobility does not reflect overall conformational changes. We computed the covariance matrix of the Cα positions and diagonalized the covariance matrix to predict the dominant motions and their corresponding magnitudes around the equilibrium structure of the BRAF:14–3–3_2_ complex. Eigenvectors and eigenvalues of the covariance matrix were calculated using ProDy software.^22^

Loop I and II structures are excluded from the principal component analysis (PCA). In the first step, each conformation in the trajectory was superimposed onto the initial structure. In the second step, a variance–covariance matrix was constructed by calculating the deviations of positions from the average structure. The elements of the covariance matrix, *C*, were calculated as

$$C_{\mathrm{ij}}=<{(x}_{i}-{<x}_{i}>)\left(x_{j}{-<x}_{j}>\right)>$$

where *x*_*i*_ and *x*_*i*_ are the coordinates of residues *i* and *j*, respectively, and the brackets denote the ensemble average taken over our conformer library. The covariance matrix, *C*, can be decomposed as

$$C=P\Delta P^{T}$$

where the eigenvectors, *P*, represent the principal components (PCs) and the eigenvalues are the elements of the diagonal matrix, Δ. Each eigenvalue is directly proportional to the variance it captures in its corresponding PC. The eigenvectors represent the intrinsic collective motions of the protein and the corresponding eigenvalues represent the magnitudes of these motions. To observe the collective motions and their magnitudes, normalized correlations of the systems were calculated as

$$C_{\mathrm{ij}}=\frac{\sum_{l=1}^{10}\frac{U_{\mathrm{il}}U_{\mathrm{jl}}}{\Omega_{\mathrm{ll}}}}{{\left(\sum_{m=1}^{10}\frac{U_{\mathrm{im}}U_{\mathrm{jm}}}{\Omega_{\mathrm{mm}}}\right)}^{1/2}{\left(\sum_{n=1}^{10}\frac{U_{\mathrm{in}}U_{\mathrm{jn}}}{\Omega_{\mathrm{nn}}}\right)}^{1/2}}$$

where *U* is the matrix of eigenvectors and Ω is the diagonal matrix of eigenvalues. The cross-correlation maps indicate the correlation of motion between different parts of the protein.

### 1D Umbrella Sampling

We manually selected six interdomain distances to serve as coordinates for computing free energy profiles using umbrella sampling:^23,24^ (1) RBD to KD, (2) RBD to 14–3–3 (S729), (3) CRD to 14–3–3 (S729), (4) KD to 14–3–3 (S729), (5) KD to 14–3–3 (S365), and (6) CRD to KD. Each interdomain distance is defined as the distance between the center of mass (COM) of every Cα atom in each domain. All 1D US simulations were performed using OpenMM (v8.0.0).^25^ The temperature was maintained at 310 K using Langevin dynamics, as implemented in the OpenMM package. For each coordinate, we divided the interdomain distances with windows spaced evenly over the intervals: (1) 2 to 8 nm, (2) 1.4 to 7.4 nm, (3) 1 to 7 nm, (4) 3 to 9 nm, (5) 3 to 9 nm, and (6) 2 to 8 nm. For the RBD to KD coordinate, we divided the target distance interval into 31 windows, while for all other distances, we used 61 windows. We restrained each simulation using a harmonic potential with 239 kcal/mol*nm force constant and minimum placed at each window center, except for the RBD-to-KD distance for which we used a force constant of 119.5 kcal/mol*nm. These force constants were selected to be as small as possible while still maintaining sufficient overlap between adjacent windows.

We equilibrate the system in each umbrella sampling window by selecting the final frame from one of the unbiased MD simulations described above and restraining the system at the initial interdomain distance. We then progressively shift the center of the restraint from the initial value to the target position by using 20 stages, starting from the initial position and ending at the target restraint position for each window. Each stage was simulated for 2.5 ns with a fixed window center before moving to the next restraint location, allowing 50 ns to equilibrate the system in each window. All production simulations were then simulated with a fixed harmonic restraint for 12 h on Frontier or Lassen supercomputing systems, resulting in ∼20–40 ns of simulation time per window. This procedure was repeated for each of the 27 loop conformers and across all interdomain distances, resulting in an aggregate 501.8 μs total simulation time. All analysis code and simulation setups were implemented in Python and Bash scripting languages.

We computed the 1D potentials of mean force (PMF) using the weighted histogram analysis method (WHAM)^23,26^ implemented in software from the Grossfield lab (v2.1.0).^26,27^ For each window, we extracted the time series of COM distances and subsampled those trajectories using the timeseries module of pymbar software^28,29^ into a set of statistically independent samples. We then aggregated the subsampled data across simulations initialized from different starting loop conformations and used this combined dataset as input to WHAM software. For each pulling direction, we computed the PMF over 40 evenly spaced bins spanning between (1) 2.5 and 9.0 nm for RBD to KD, (2) 1.5 and 8 nm for the RBD to 14–3–3 (S729), (3) 1.5 and 7.0 nm for the CRD to 14–3–3 (S729), (4) 3.5 and 7.5 nm for 14–3–3 (S729) to KD, (5) 3.5 and 9.0 nm for 14–3–3 (S365) to KD, and (6) 2.5 and 9.0 nm for CRD to KD. We estimated the variance of each free energy profile using the bootstrapping feature of the WHAM code using 100 independent MC trials.

### 2D Metadynamics Simulations

Metadynamics is an enhanced sampling technique designed to investigate physical phenomena that occur at time and length scales beyond the reach of standard MD simulations.^30,31^ This method enhances the sampling of rare events by adding a history-dependent bias energy into the system’s Hamiltonian. This bias energy takes the form of a Gaussian potential, as defined by

$$V_{G}\left(s,t\right)=\int_{0}^{t}{dt}^{\prime }\;w\;\exp\left[-\sum_{i=1}^{d}\frac{(S_{i}\left(R\right)-S_{i}(R{\left(t^{\prime }))\right)}^{2}}{2\sigma_{i}^{2}}\right]$$

where σ represents the width of the Gaussian for the *i*th collective variable (CV), *w* is the height of the Gaussian, and *S*_i_(*R*(*t*′)) denotes the value of the *i*th CV at time *t*′.

By continually adding these Gaussian potentials, metadynamics modifies the energy landscape of the system, effectively accelerating the sampling of rare events. This bias discourages the system from revisiting previously explored regions in the CV phase space, promoting more thorough exploration and ideally leading to ergodic and diffusive motion along the chosen CVs.

As the simulation progresses and the bias potentials accumulate, the system is driven to explore new configurations. The sum of the negative bias energies added during the simulation provides an estimate of the underlying free energy profile along the selected CVs. This free energy profile is a powerful tool for understanding the thermodynamics and kinetics of the system, offering insights into the mechanisms of complex processes that are otherwise difficult to capture with standard MD simulations.

Here, metadynamics simulations spanning ∼90 ns (15 replicas) were carried out using an approach similar to the one-way sampling approach described previously^32^ to determine the free energy surfaces for the 14–3–3 interaction with BRAF when RBD is bound or unbound. The total simulation time for each case is 1.35 μs. The simulations were started after the initial equilibration using the same parameters described above. All simulations were performed using the GROMACS 2019.6 package patched with the PLUMED 2.6.6 code.^33–35^

The CVs were selected to describe the motion of the 14–3–3 protomer with the S729 site. The use of two CVs has been shown to provide more stable results than the use of one CV.^36^ The first CV (d_1_COM) is the distance between the COM of the first half of 14–3–3 (S729) (the region closer to CRD) and CRD, whereas the second CV (d_2_COM) is the distance between the COM of the second half of 14–3–3 (S729) (the region closer to RBD) and CRD. The Gaussian bias energy is deposited every 1000 steps with a height of 0.05975 kcal/mol and width of 0.08 nm for the CVs that describe d_1_COM and d_2_COM.

## Supplementary Material



## Data Availability

Simulation input and parameter files, analysis scripts, and starting and final protein structures for all molecular dynamics simulations are publicly available for download at https://bbs.llnl.gov/data. Any additional information including complete simulation trajectories is available upon request from the corresponding authors (tempkin1@llnl.gov and aydin1@llnl.gov).

## Notes

ATP binding site in KD, RAF loop placement and relaxation, extent and equilibration of the RAF loop Configurations, details on the PCA analysis of the BRAF:14-3-3_2_ complex, and details on the domains/residues in the BRAF:14-3-3_2_ model (PDF)

## ∇

BridgeBio Oncology Therapeutics, South San Francisco, California 94080, USA

## ○

Global Drug R&D Center, Huadong Medicine, Hangzhou 310011, P. R. China

## Abbreviations

MD: molecular dynamics
AA: all-atom
PCA: principal component analysis
PDB: protein data bank
HPC: high-performance computing
cryo-EM: cryo-electron microscopy
RBD: RAS-binding domain
CRD: cysteine-rich domain
KD: kinase domain
CVs: collective variables
