# Supplementary material for: Free Energy and Flexibility Analysis of Autoinhibited Human BRAF
Source: J Chem Inf Model. 2026 Apr 21;66(9):5372–81. doi: 10.1021/acs.jcim.5c03126 (PMC13169344; doi:10.1021/acs.jcim.5c03126)
Supplement: Supplementary file 1 [file ci5c03126_si_002.pdf]

Supporting Information

Free energy and flexibility analysis of autoinhibited human BRAF

Jeremy O. B. Tempkin[1,‡,], Fikret Aydin[1,‡,*], Sebnem Essiz[2], Yue Yang[1,†], Timothy S. Carpenter[1], David E. Durrant[3], Deborah K. Morrison[3], Helgi I. Ingólfsson[1], Frederick H. Streitz[4], Dwight V. Nissely[5], Felice C. Lightstone[1], Xiaohua Zhang[1,§]*

[1]Physical and Life Sciences Directorate, Lawrence Livermore National Laboratory, Livermore, CA 94550, USA

[2]Faculty of Natural Sciences and Engineering, Department of Molecular Biology and Genetics, Kadir Has University, 34083 Istanbul, Turkey

[3]Laboratory of Cell and Developmental Signaling, Center for Cancer Research (CCR), National Cancer Institute (NCI), Frederick, MD 21702, USA

[4]Computing Directorate, Lawrence Livermore National Laboratory, Livermore, CA 94550, USA

[5]NCI RAS Initiative, Cancer Research Technology Program, Frederick National Laboratory for Cancer Research, Frederick, MD 21702, USA

‡These authors contributed equally.

*Corresponding Authors: Jeremy O. B. Tempkin, tempkin1@llnl.gov; Fikret Aydin, aydin1@llnl.gov

Present Addresses

†BridgeBio Oncology Therapeutics, South San Francisco, CA 94080, USA

§Global Drug R&D Center, Huadong Medicine, Hangzhou 310011, P. R. China

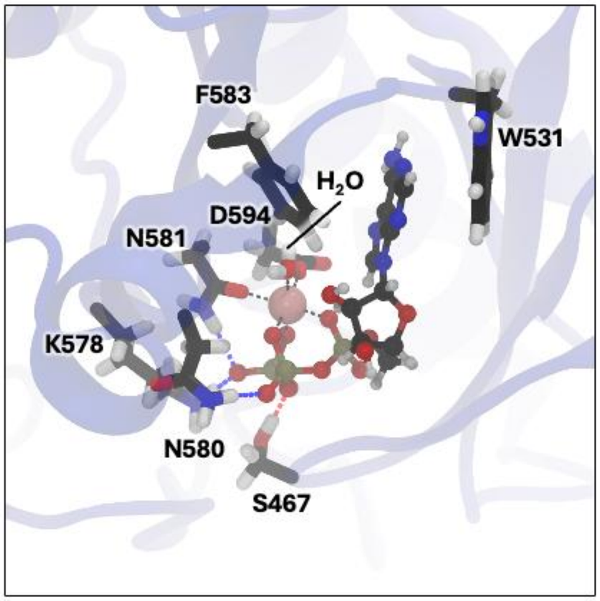

FIGURE S1. ATP binding site in KD. ATP and its coordinating Mg^{2+} ion were placed using MOE. ATP-Mg initial location and contacting amino acids were based on PDB 6U2G (chain B) followed by a short energy minimization to relax the ATP-Mg complex. The image shows the KD (blue), focused on the ATP binding pocket highlighting the ATP, Mg^{2+} (pink sphere) and contacting residues.

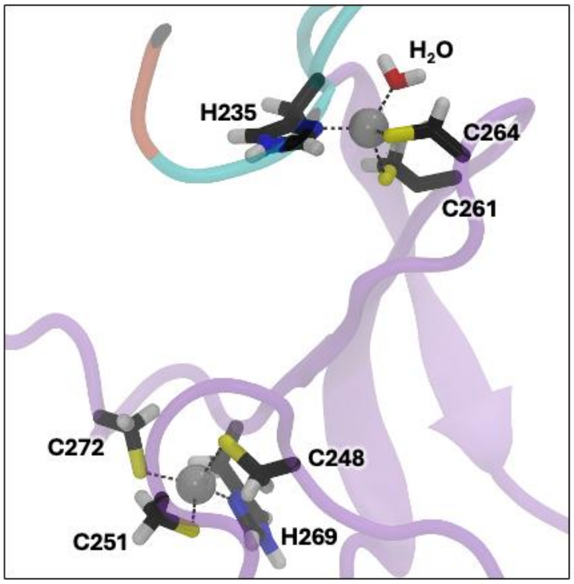

FIGURE S2. Zinc coordination sites in CRD. The reference structure PDB 7MFD does not resolve the fourth coordination of one of CRD's zinc (top). We completed the coordination of that zinc by adding a coordinating water molecule and placed it to ensure proper coordination of residue H235. The image shows the CRD's two zinc finger motifs highlighting the Zn^{2+} atoms and the contacting residues. The CRD is shown in purple, the C-terminal end of the RBD in orange, and the RBD-CRD linker in cyan. The two Zn^{2+} ions are shown in grey spheres.

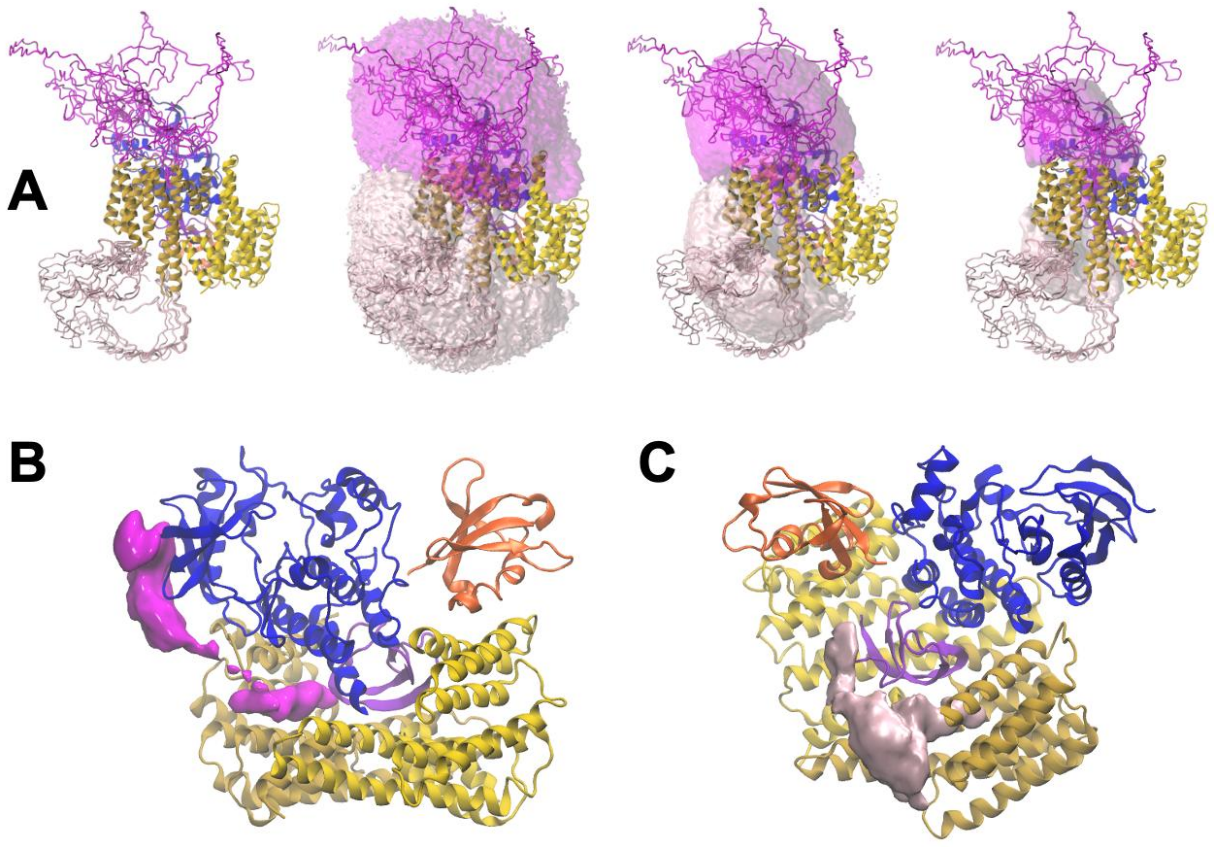

FIGURE S3. RAF loop placement and relaxation. Loops I (pink, residues 274 to 359) and II (magenta, residues 371 to 448) are disordered and are built into the model. The loops are built in with MOE and simulations are started with 27 different loop initial configurations, panel A. In the simulations the loops are very dynamic, sampling a larger confirmational space with the overall extent somewhat reduced compared to the initial placement, see example radius of gyration Fig. S4. A) The loops extended and common location are indicated with transparent loop density maps, showing low, medium, and high isovalues to illustrate the extended, commonly occupied, and frequently occupied areas for the loops. B) and (C) show zoomed in views of very high loop density areas for loop II and I, respectively, corresponding to where the loops are attached to the protein complex.

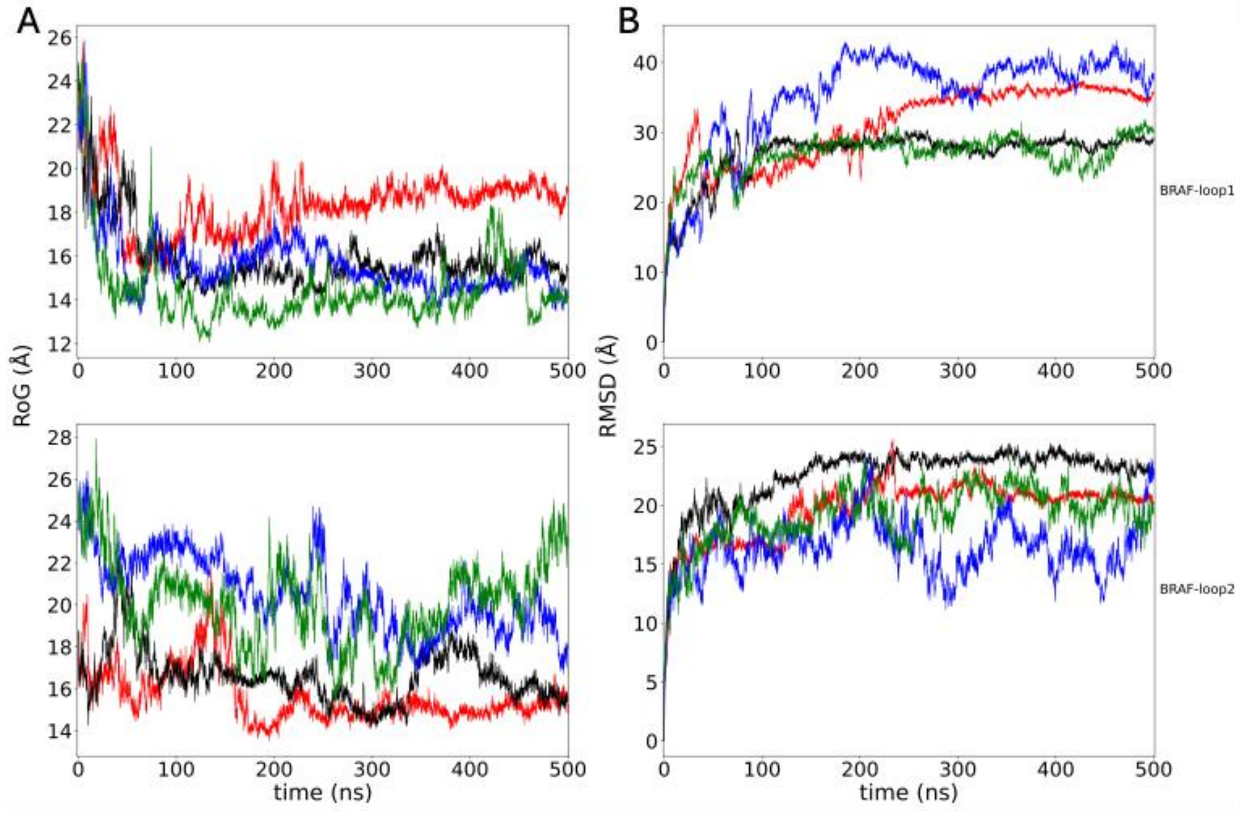

FIGURE S4. Extent and equilibration of the RAF loop configurations. Radius of gyration (RoG) (A) and RMSD (B) is shown for four random simulations of the 135 unbiased MD simulations. Top plots are for loop I (residues 282 to 359) and bottom plots for loop II (residues 371 to 456).

A

	Fraction of Variance	Collectivity
Mode 1	0.24	0.42
Mode 2	0.12	0.37
Mode 3	0.06	0.59
Mode 4	0.05	0.62
Mode 5	0.05	0.11
Mode 6	0.04	0.26
Mode 7	0.03	0.14
Mode 8	0.03	0.34
Mode 9	0.03	0.1
Mode 10	0.02	0.14

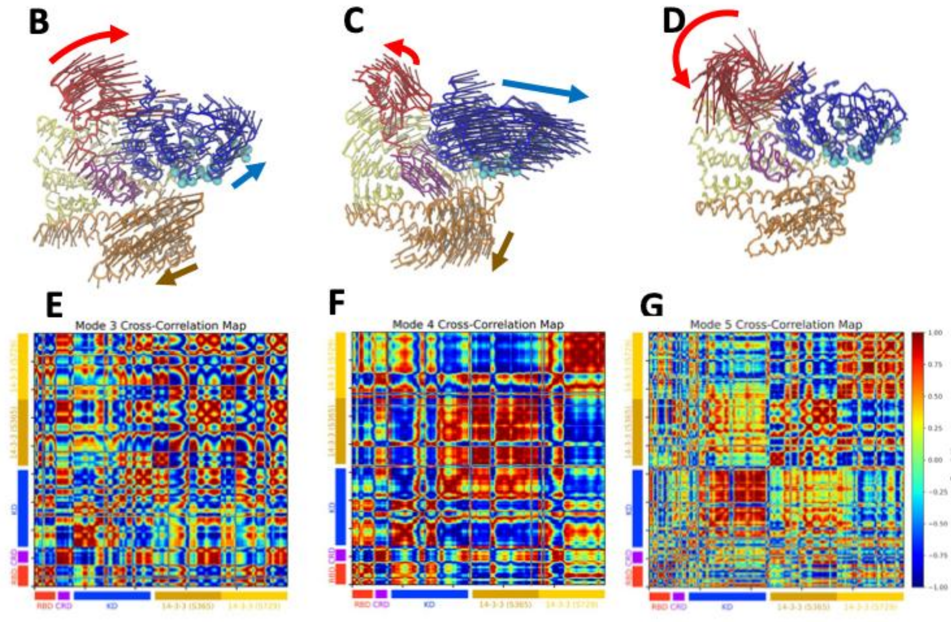

Figure S5. Additional modes of PCA analysis of the autoinhibited BRAF:14-3-32 complex. A. Fractional contribution and collectivity of 10 PCA modes. The proteins/protein-domains are shown in RBD-red, CRD-purple, KD-blue, 14-3-3 (S365)-gold, 14-3-3 (S729)-yellow. Cyan spheres display kinase-kinase dimer interface residues B) Shows the 3rd PCA mode visualization and (C) the cross-correlation map of the mode. C) and F) show the same for the 4th PCA mode. D) and G) show the same for 5th PCA mode. The colored arrows indicate the primary motions of each mode.

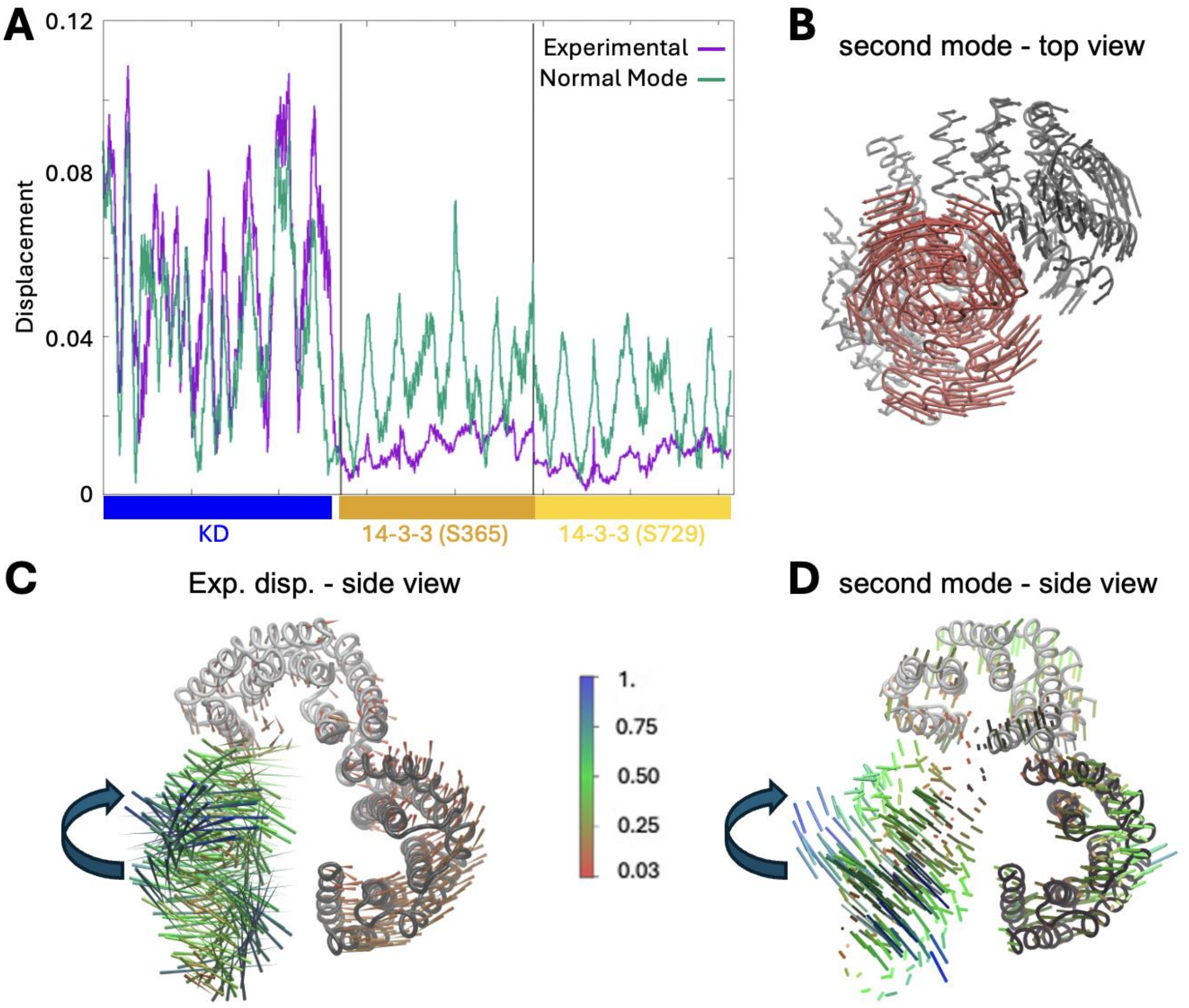

Figure S6. Details on the PCA analysis of the BRAF:14-3-3_2 complex. A) Comparison of the best overlapping mode (second PCA mode) with experimental conformational displacement. B) Displacement according to computed second mode, top view. C) Displacement of 14-3-3_2 and KD according to experimental conformational change PDB 7MFF to 7MFD. D) Displacement according to the computed second PCA mode. To assess whether the conformational flexibility around the autoinhibited state is related to the conformational changes required for the KD to transition between the monomeric and dimeric states, we compared these motions to the structural differences apparent between the dimerized BRAF (PDB: 7MFF) and monomeric BRAF (PDB:

7MFD) cryo-EM structures. We first superimposed the KD and 14-3-3 dimer from the monomeric BRAF structure 7MFD onto the KD and 14-3-3_2 from the dimeric structure 7MFF. The RBD and CRD segments are discarded from 7MFD structure as well as from the PCA modes as these domains are not resolved in the dimer structure. Then we calculated the unit normalized displacement vector between 7MFD and 7MFF and compared the linear displacement required to transform the KD and 14-3-3_2 complex. We show that the similarity of the vector is checked against the largest amplitude PCA modes. The second mode has a rotation type of movement for kinase. This second mode displayed highest similarity (dot product of the two vectors is -0.44) to the conformational change in between the cryo-EM structures. In panel A we show the experimental displacement vector together with the second PCA mode; 14-3-3_2 domain is more mobile in the PCA mode, while the KD region displacements seem similar. In panel B the same PCA vector is projected onto the structure with arrows from top view of the rotation axis of the kinase. The rotational motion of kinase around the same axis is visible in experimental displacement in between 7MFD and 7MFF (panel C) and the second mode obtained from the PCA analysis of the MD trajectory (panel D).

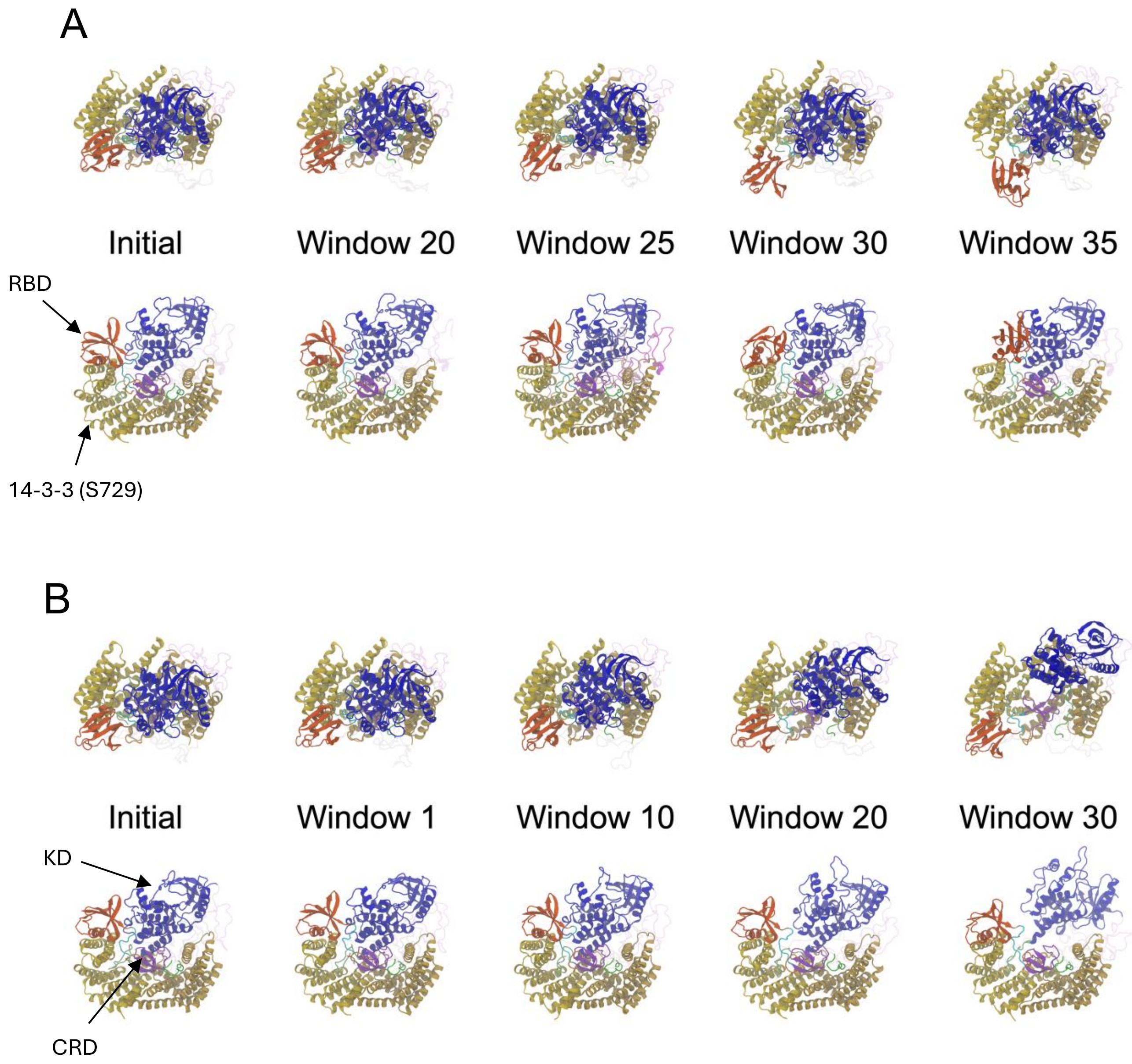

Figure S7. Snapshots of the BRAF complex taken from 1D umbrella sampling windows along the RBD to 14-3-3 (S729) pulling coordinate (panel A) and the KD to CRD pulling coordinate (panel B). A) Snapshots from the RBD to 14-3-3 (S729) pulling coordinate including the fully autoinhibited structure (initial) and a snapshot sampled from windows 20, 25, 30 and 35. Second row shows the structures rotated by 90 degrees. B) Snapshots from the KD to CRD pulling

coordinate including the fully autoinhibited structure (initial) and a snapshot from windows 1, 10, 20 and 30. Second row shows the structures rotated by 90 degrees.

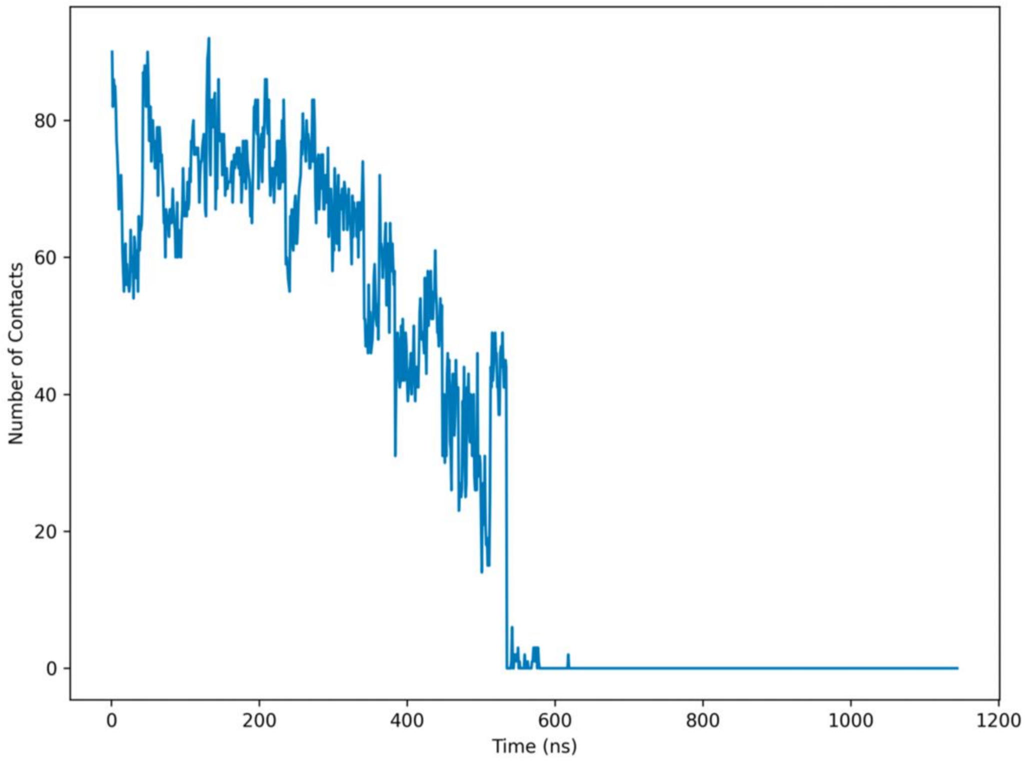

Figure S8. The number of contacts between RBD and 14-3-3 interface as a function of time during the US simulation involving RBD-14-3-3 distance collective variable. The number of inter-residue contacts between the RBD and 14-3-3 was computed for every frame across all biased simulation windows. Two residues were defined to be in contact when the distance between their Cα atoms was less than 10 Å. The contact counts obtained from individual windows were then concatenated in order to generate a continuous time series, providing the number of contacts as a function of time.

Table S1. Details on the domains/residues in the BRAF:14-3-3_2 model. Column 1 lists the names of each domain/region. Columns 2 and 3 list the starting and ending residue numbers for each region respectively. Column 4 describes whether the position of those residues were determined from the cryo-EM structure (PDB: 7MFD) or added to the structure by the modeling approaches described in the main text.

Domain/region	Start residue	End residue	Cryo-EM or modeled?
RBD	156	201	Cryo-EM
	202	*203*	*Modeled*
	204	227	Cryo-EM
RBD-CRD linker	*228*	*234*	*Modeled*
CRD	235	273	Cryo-EM
CRD-zinc loop	*274*	*281*	*Modeled*
Loop I	*282*	*359*	*Modeled*
pS365 region	360	370	Cryo-EM
Loop II	*371*	*448*	*Modeled*
	449	456	Cryo-EM
KD	457	723	Cryo-EM
PS729 region	724	738	Cryo-EM
14-3-3 zeta (S365)	2	69	Cryo-EM
	70	*72*	*Modeled*
	73	203	Cryo-EM
	204	*209*	*Modeled*
	210	230	Cryo-EM
14-3-3 zeta (S729)	2	69	Cryo-EM

	70	*71*	*Modeled*
	72	133	Cryo-EM
	134	*136*	*Modeled*
	137	230	Cryo-EM
